# The Parallel World of Dyspnea: A Case Report

**DOI:** 10.7759/cureus.54065

**Published:** 2024-02-12

**Authors:** Flávia Baduy, Lenia F Costa, Sofia L Ferreira, Frederico Rocha, Avelina Moniz

**Affiliations:** 1 Tejo Family Health Unit, São José Local Health Unit, Loures, PRT

**Keywords:** clinical case report, diagnostic reasoning, autoimmune disease, clinical thinking, respiratory distress

## Abstract

Dyspnea can be found as a symptom of a wide range of diseases. Clinical thinking usually leads us to more common or frequent syndromes and diseases. This case report alerts us to keep investigating when faced with therapeutic failure or the arising of new symptoms. The subject in this case had dyspnea as an initial presentation of his disease and was treated initially as a case of heart dysfunction. Nevertheless, because his symptoms did not respond to the treatment and even got worse, he was sent to the emergency room where he was medicated and discharged with the same diagnostic hypothesis. In light of a new characteristic symptom - ptosis - the hospital team expanded its clinical and laboratory investigation to neuromuscular diseases, reaching out the diagnosis of myasthenia gravis.

## Introduction

Dyspnea can be defined as the subjective feeling of painless, although uncomfortable, shortness of breath, consisting of qualitatively distinct sensations that vary in intensity, usually leading the patient to seek medical care [1,2]. Notably, dyspnea is a symptom and must be distinguished from signs, such as tachypnea or the use of accessory muscles. There are multiple possible causes: increased chemical or psychological drive, increased breathing work (by obstruction or restriction), neuromuscular imbalance, or even a mixture of all of these, and the result of these can be reflected in certain characteristics described by the patient [2]. Additionally, the psychological component cannot be ignored because, as a subjective feeling, factors such as the patient’s past experiences and emotions may contribute to different perceptions of dyspnea [2,3]. Based on this knowledge, when a patient came to our office and reported dyspnea, we were typically directed to some differential diagnoses and considered the most common and frequent diseases and syndromes in our region.

Our clinical thinking encouraged us to review the comorbidities and family history to reach a diagnosis. However, a clinician must note that diagnosis is a process that must be reviewed when faced with new facts. Therefore, the main goal of this case report is precisely to alert primary care physicians to not underestimate such common complaints, especially in patients who already have comorbidities suggestive of pulmonary or myocardial diseases, for example.

## Case presentation

The patient is a 75-year-old man, Caucasian, retired from administrative service, married, independent in basic and instrumental activities of daily living, and socioeconomic level of class III (based on the Graffar scale [4]). He has had a personal history of type 2 diabetes mellitus, arterial hypertension, dyslipidemia, and insomnia for several years (without being able to specify the date of the diagnoses) cataracts, and moderate to severe retinopathy since 2018. The patient denies harmful habits, such as smoking or recreational drugs, and any previous surgeries, hospitalizations, accidents, or known allergies. His usual medication included omeprazole 20 mg, ramipril 5 mg, metformin 850 mg, gliclazide 60 mg, pravastatin 40 mg, alprazolam 1 mg, and latanoprost 0.05 mg/mL.

The patient visited his family doctor (FD) because of orthopnea and tiredness five days prior. Anamnesis revealed dyspnea with mild/medium efforts (Modified Medical Research Council (mMRC) grade 2), a calculated risk-adjusted model for individuals aged over 70 years without preexisting cardiovascular disease (Systematic Coronary Risk Evaluation 2-Older Persons; SCORE2-OP) of 21%, and sustained apyrexia. Auscultation detected mild bilateral fevers and hypophonetic heart sounds without edema in the lower limbs. When FD considered the hypothesis of heart failure, the medication was altered to include furosemide 40 mg and bisoprolol 1.25 mg, and a requisition was made for an electrocardiogram (ECG) and chest X-ray.

The patient returned a week later, reporting worsening dyspnea, which, at this point, forced him to stop after a few minutes of walking. He showed normal exams, ECG with sinus rhythm, left bundle branch hemiblock, that was already known, and no signs of acute ischemia. At this point, the FD referred the patient to the emergency room (ER) for the first time. At the ER, blood tests revealed troponin I 11.7 ng/mL (reference range: <0.04 ng/mL), D-dimers 418 mcg/L (reference range: <500 mcg/L), and NT-ProBNP <50 pg/mL (reference range: <879 pg/mL); a new chest X-ray showed no pleural effusion or signs of pulmonary condensation; and an echocardiogram revealed a slightly dilated aortic root and ascending aorta, slight degenerative alterations of the mitral and aortic valves, without hemodynamic compromise. The patient received a readjusted therapy for heart failure, with addiction to spironolactone 25 mg and an increased dose of ramipril to 10 mg, and then was discharged for follow-up by his FD.

**Figure 1:**
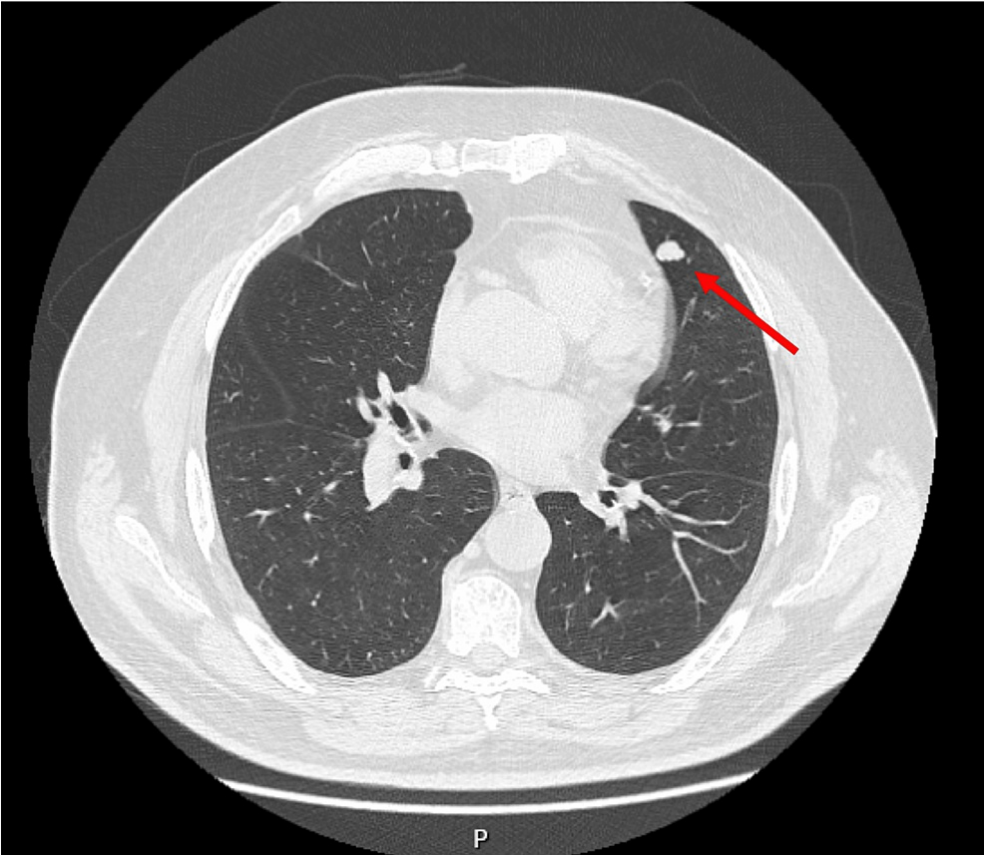
Chest CT with a pulmonary nodule detected.

After an initial improvement, the patient experienced a resurgence of symptoms two days after being discharged from the ER, such as headaches, blurred vision, dizziness when standing upright, tiredness when chewing, and neck drooping because of weakness throughout the day, which induced him to look for his FD again. Because of the new symptoms, he was sent by his FD to his second visit to the ER on the same day. An initial chest CT detected a pulmonary nodule (Figure 1) with no other relevant findings, in particular excluding a possible thymoma, which led the physicians to hospitalize him in an inpatient study for a possible paraneoplastic syndrome.

In view of the apparent dysphagia, the otorhinolaryngologist observed the patient and reported no alterations in the oropharynx and larynx. Cranial nerve tests also showed no abnormalities.

**Figure 2:**
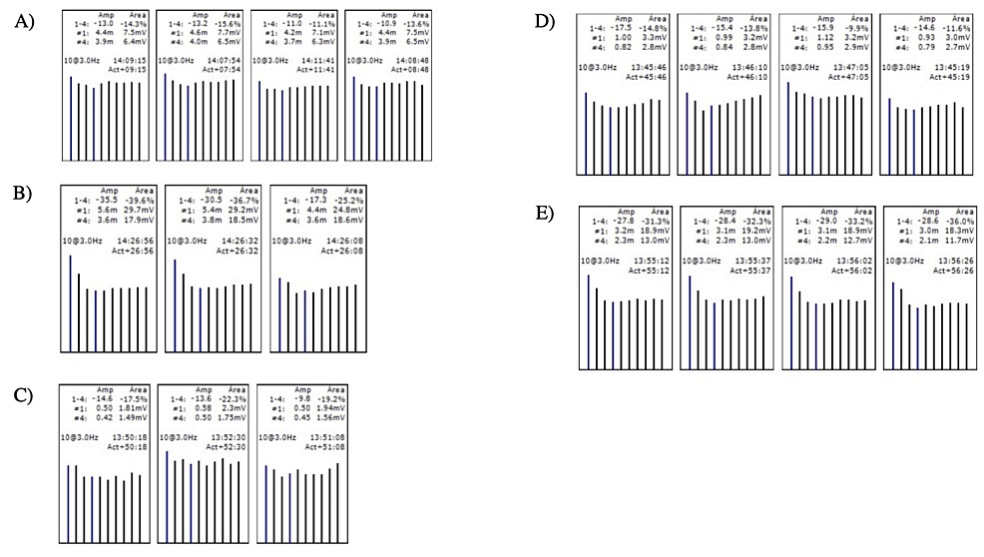
Electromyography of the (A) left anconium muscle; (B) left deltoid; (C) left frontalis; (D) left nasalis; and (E) left trapezius. A) Left anconium muscle: decrease in rest from 10.9 to 13.2% in range and from 11.1 to 15.6% in area. B) Left deltoid muscle: decrease in rest from 17.3 to 35.5% in range and from 25.2 to 39.6% in area. C) Left frontalis muscle: decrease in rest from 9.8 to 14.6% in range and from 17.5 to 22.3% in area. D) Left nasalis muscle: decrease in rest from 14.6 to 17.5% in range and from 9.9 to 14.8% in area. E) Left trapezius muscle: decrease in rest from 27.8 to 29.0% in range and from 31.0 to 36.0% in area.

During hospitalization, a new symptom appeared: ptosis. Considering this new symptom, the doctors referred the patient to the neurology service, where a neurologist performed an electromyography. The neurophysiological study documented a postsynaptic neuromuscular transmission defect in the left frontalis, nasalis, trapezius, deltoid, and anconeus muscles (Figure 2), which supports the clinical diagnosis of myasthenia gravis (MG), generalized form, with no neurophysiological evidence of presynaptic end-plate disease.

**Figure 3:**
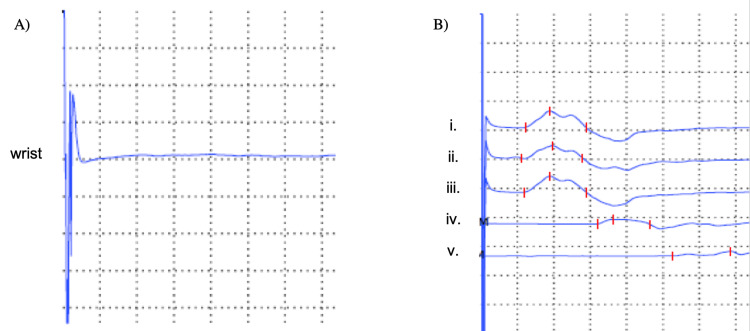
Electromyography of the (A) right sensory ulnaris nerve and (B) right motor ulnaris nerve. A) Right sensory ulnaris nerve: lack of response. (B) Right motor ulnaris nerve: slight slowing of the motor conduction speed in the forearm and marked slowing of the motor conduction speed in the elbow; slightly prolonged distal latency; very reduced distal amplitude; partial conduction block to nerve stimulation below the elbow; no ease after short-term exercise. (i. wrist-r-range of movement; ii. wrist-e-range of movement; iii. wrist range of movement; iv. below the elbow range of movement; v. above the elbow range of movement).

Additionally, the neurophysiological study also showed a right cubital neuropathy, located at or below the elbow, with mixed pathophysiology (axonal and demyelinating) and high severity, resulting in a canal syndrome of this nerve (Figure 3).

During new blood tests, they detected positive anti-acetylcholine antibodies (>8.0 nmol/L - reference range <0.05 nmol/L), corroborating the diagnosis of MG. Antibodies reactive to the striated muscle (Str-Abs) were negative, excluding thymomatous MG or thymic disease, as well as antibodies against muscle-specific tyrosine kinase (MuSK-Abs).

Concerning the pulmonary nodule, a positron emission tomography scan was done 15 days after his admission, which did not reveal any abnormal metabolism.

**Figure 4:**
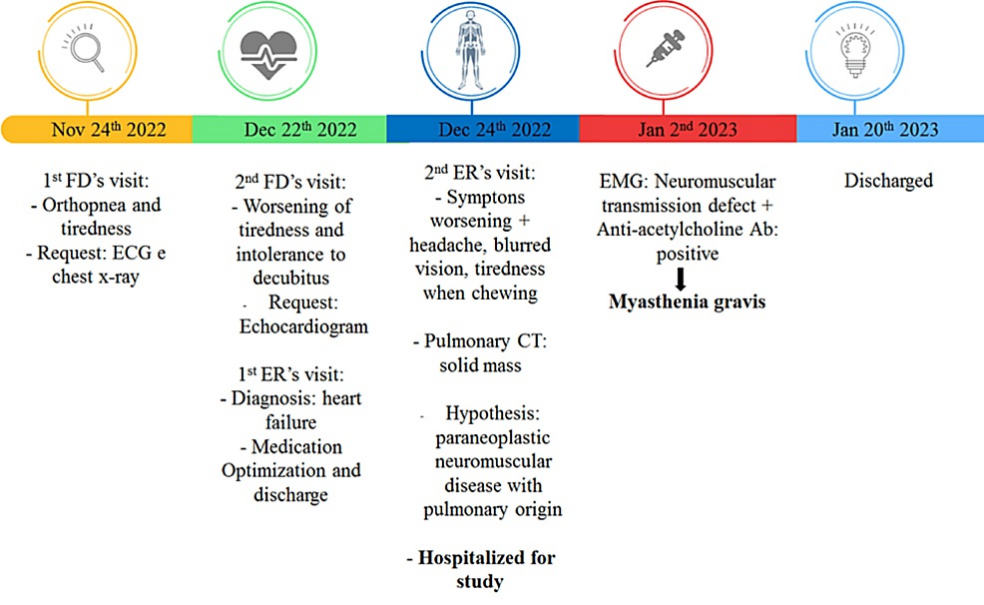
Timeline of diagnosis. FD: Family doctor; ER: emergency room, EMG: electromyography.

From the neurological perspective, the patient started therapy with intravenous immunoglobulin over five days, prednisolone 60 mg/day, and pyridostigmine 60 mg/3id with clinical improvement. We summarize the timeline of the diagnosis in Figure 4.

## Discussion

MG is a chronic autoimmune disease, characterized by easy tiredness and episodic muscle fatigue, localized or generalized, of neurological origin [5]. Dyspnea is rarely the first symptom of MG; rather, ptosis or diplopia are the initial symptoms in 60% of patients. This is followed by difficulty chewing, swallowing, or talking in 16% of patients and limb weakness in 10% of patients [5]. Patients with respiratory weakness may be severely dyspneic, hypoxic, or hypercarbic, without evidence of tachypnea, use of accessory muscles, or distress [5,6].

Breathlessness appears in a wide range of situations, such as a sedentary lifestyle and psychosocial stress. Nevertheless, it is an important symptom to consider in organic pathologies such as heart failure, chronic obstructive pulmonary disease, or cancer. As such, it has been associated with poor quality of life and survival, usually leading the patient to seek healthcare at an ER or with their FD [7]. Therefore, some patients end up delaying seeking medical help because of neglecting their symptoms, health illiteracy, or lack of timely access to healthcare [8].

This clinical case highlights the importance of a careful anamnesis and physical examination, even in the face of symptoms and signs easily related to more common pathologies. Moreover, for cases of persistent dyspnea, even with improved therapeutic, we suggest a full blood count and spirometry, chest X-ray, and ECG as appropriate as clinical assessment by primary care physicians [9]. However, it is important to keep in mind differential diagnoses when the patients seek a second visit to their FD, owing to suboptimal symptom control, as this can create an overuse of pharmaceuticals and their possible side effects [9].

Although some conditions are rare, our clinical thinking must be strengthened through studies on differential diagnoses, until most of the clinical and laboratory criteria are exhausted and met. Primary healthcare doctors must avoid remaining inertia and pursue viable treatment options and available tests, obviously always counting on the referral and collaboration of hospital medical specialties. We emphasize the great importance of FDs continuously updating their knowledge, with a focus on the differential diagnoses of frequent symptoms, symptom assessment, follow-up, referral, and patient education for warning signs. Even knowing that the appointment time for FDs is often short, common signs and symptoms should never be overlooked or ignored.

## Conclusions

The main goal of this case report is to discuss and alert FDs and other primary care professionals to keep in mind that it is necessary to be proficient beyond the obvious and the common. They should also remember that in the primary care context, there are a multitude of noninvasive tests and procedures (such as spirometry) that can be used as an initial approach or when things do not go as expected. Diagnosis is a process that must always be reviewed using our clinical thinking with evidence-based medicine.
